# Molecular Basis of Bone Aging

**DOI:** 10.3390/ijms21103679

**Published:** 2020-05-23

**Authors:** Addolorata Corrado, Daniela Cici, Cinzia Rotondo, Nicola Maruotti, Francesco Paolo Cantatore

**Affiliations:** Rheumatology Clinic, Department of Medical and Surgical Sciences, University of Foggia, 71122 Foggia, Italy; daniela.cici@gmail.com (D.C.); cinzia.rotondo@gmail.com (C.R.); nicola.maruotti@unifg.it (N.M.); francescopaolo.cantatorer@unifg.it (F.P.C.)

**Keywords:** osteoporosis, bone aging, bone loss, senescence

## Abstract

A decline in bone mass leading to an increased fracture risk is a common feature of age-related bone changes. The mechanisms underlying bone senescence are very complex and implicate systemic and local factors and are the result of the combination of several changes occurring at the cellular, tissue and structural levels; they include alterations of bone cell differentiation and activity, oxidative stress, genetic damage and the altered responses of bone cells to various biological signals and to mechanical loading. The molecular mechanisms responsible for these changes remain greatly unclear and many data derived from in vitro or animal studies appear to be conflicting and heterogeneous, probably due to the different experimental approaches; nevertheless, understanding the main physio-pathological processes that cause bone senescence is essential for the development of new potential therapeutic options for treating age-related bone loss. This article reviews the current knowledge concerning the molecular mechanisms underlying the pathogenesis of age-related bone changes.

## 1. Introduction

Bone is a complex, metabolically active and constantly modifying tissue. Apart from the essential mechanical properties consisting in the protection of internal organs, the support of soft tissues and locomotion, bone tissue exerts a wide variety of metabolic functions [1], as it plays an essential role in systemic mineral homeostasis and it is involved in hematopoiesis due to the close relationship between bone cells and hematopoietic bone marrow cells [2].

An impairment of the entire bone functions is observed with aging, resulting in changes of the structural and geometrical characteristics of the skeleton, reduced bone mass, reduced load-bearing capacity, altered response to systemic humoral factors and a decline in reserve of mineral contents. Taken together, these changes result in osteoporosis and an increased risk of fractures [3]. Osteoporosis is a systemic bone disease characterized by low bone mass and the micro-architectural deterioration of bone tissue, leading to enhanced bone fragility and a consequent increase in fracture risk [4]. Depending on the underlying etiology, osteoporosis can affect all ethnic, gender and age groups, although it is most frequently observed in post-menopausal women and in older people of both sexes, and it represents a major cause of morbidity and disability in the elderly population [5]. Age is an independent risk factor of fracture: older subjects present up to a 10-fold-increase in fracture risk over ten years compared with younger subjects.

**Figure 1:**
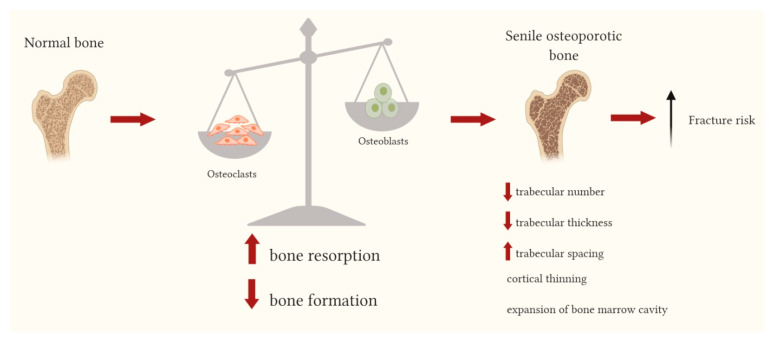
Bone changes leading to senile osteoporotic bone. With aging an imbalance in bone remodeling phases is observed with an increased bone resorption (initiated by osteoclasts) and a decrease in bone formation (carried out by osteoblasts). This imbalance leads to both trabecular and cortical alterations: the reduction of the trabecular number, the decreased trabecular thickness and the increased trabecular spacing; the cortical thinning and the expansion of bone marrow cavity. ↑: increased; ↓: decreased.

Post-menopausal osteoporosis, in which pathogenesis the estrogen deficiency plays an essential role, mainly affects trabecular bone, while age-related bone changes occur in both the trabecular and cortical bone. In trabecular bone the main alterations are represented by the reduction of the trabecular number, decreased trabecular thickness and increased trabecular spacing, whereas in cortical bone, cortical thinning and the expansion of bone marrow cavity, due to increased endocortical resorption and increased bone formation on the periosteal surface, are observed (Figure 1). The mechanisms of age-related changes in bone tissue are very complex and implicate systemic and local factors; increased fracture risk related to bone aging is determined by the combination of alterations occurring at cellular, tissue and structural levels, whose multi-factorial pathogenesis involves genetic factors, reduced cell differentiation, altered responses of bone cells to several biological signals and to mechanical loading [6,7].

## 2. Adult Bone Cells

Bone is a composite tissue, consisting of inorganic mineral crystals and organic components represented by bone cells, bone marrow cells, extracellular matrix proteins, lipids and water. Bone cells are osteoblasts, osteocytes and osteoclasts which play a key role in maintaining bone homeostasis and bone remodeling processes; their metabolic activity is regulated by a plethora of local and systemic stimuli, including mechanical, hormonal and immunological factors.

**Figure 2:**
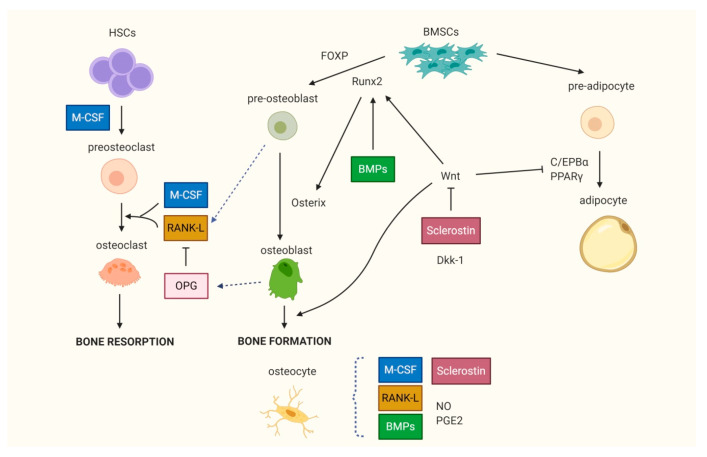
Bone cells differ0entiation. The differentiation of bone marrow stem cells (BMSCs) into osteoblasts is led by transcription factors Runt-related transcription factor 2 (Runx2), Osterix and is enhanced by Wnt, which in turn inhibits adipogenesis. The recruitment, differentiation and activity of osteoclasts are mainly regulated by the Receptor Activator of NF-κB (RANK)/RANK ligand (RANK-L)/osteoprotegerin (OPG) system and by macrophage-colony stimulating factor (M-CSF). OPG is the decoy receptor of RANK-L that strongly inhibits osteoclast formation and activity. Osteocytes are involved in the regulation of bone metabolic activities via the production of several factors. HSCs: hematopoietic stem cells; FOXP: forkhead transcription factor P; BMPs: bone morphogenic proteins; Dkk-1: Dickkopf-1; C/EBPα: CCAAT-enhancer binding protein α; PPARγ: peroxisome proliferator-activated receptor γ; NO: nitric oxide; PGE2: prostaglandin E2.

Osteoblasts and osteocytes originate from bone mesenchymal stem cells (BMSCs), whereas osteoclasts derive from the monocyte/macrophage cell line of hematopoietic stem cells (Figure 2).

Bone cells produce and remodel the mineralized extracellular matrix, whose organic component is represented mainly by type I collagen and other collagenous and non-collagenous proteins, while the inorganic component essentially consists of hydroxyapatite crystals [1]. Osteoblasts synthesize a new bone matrix and regulate the mineralization processes. Wnt signaling is the major pathway that promotes osteoblast differentiation, proliferation, maturation and activity and it plays a fundamental role in bone development and repair. The Wnt pathway is implicated in several biological processes, such as cellular proliferation and differentiation, tissue homeostasis and regeneration, embryonic development and stem cell commitment. It has been proven that the dysregulation of Wnt signaling is involved in the pathogenesis of cancer, vascular disorders, autoimmune diseases and cell senescence. Wnt signaling pathways have been classified in two main types, the canonical Wnt signaling and the non-canonical Wnt signaling, depending on the nature of the ligands and the downstream events. The canonical Wnt pathway is dependent on β-catenin intracellular levels and is mainly involved in the regulation of osteoblast differentiation, proliferation and metabolism, mineralization process and in the modulation of bone formation. When canonical Wnt signaling is inactivated, the intracellular β-catenin levels are low as it is embedded in the “destruction complex” which is an intracellular binding complex, composed of glycogen synthase kinase-3β (GSK3β), caseine kinase I (CKI), adenomatous polyposis coli (APC), and Axin. β-catenin is phosphorylated by GSK3 and subsequently subjected to degradation through the ubiquitin–proteasome pathway. GSK3 and CKI inactivate the cytosolic β-catenin by phosphorylation; Axin acts as a scaffold to the β-catenin destruction complex, promoting β-catenin degradation and thus inhibiting Wnt signaling. The canonical Wnt signaling is activated by the low-density lipoprotein receptor related proteins (LRP)-5 and LRP-6 that complexify with binds to the transmembrane Frizzled co-receptors and then stabilize the cytosolic β-catenin blocking its phosphorylation and degradation thus allowing its translocation into the nucleus, where it promotes the transcription of several targeted genes involved in osteoblast differentiation and bone formation [8,9]. Several antagonists of the Wnt pathway, including Dickkopf-1 (Dkk-1) and sclerostin are able to suppress Wnt signaling in osteoblasts. Wnt signaling enhances the expression of genes involved in the differentiation of BMSCs into osteoblasts, while it inhibits adipogenesis by the stimulation of Runt-related transcription factor 2 (Runx2) and the inhibition of CCAAT-enhancer binding protein α (C/EBPα) [10].

Osteoclasts are large, multinucleated cells derived from mononuclear cell precursors of the monocyte/macrophage lineage present in bone marrow, whose function is to resorb bone. In recent years it has been demonstrated that the recruitment, differentiation and activity of osteoclasts are mainly regulated by the receptor activator of the NF-κB (RANK)/RANK ligand (RANK-L)/osteoprotegerin (OPG) system and by macrophage-colony stimulating factor (M-CSF). RANK-L, a member of the tumor necrosis factor (TNF) superfamily, highly expressed in stromal/osteoblastic cells and activated T lymphocytes, directly induces osteoclast differentiation by binding to its receptor RANK placed on osteoclast precursor surface. The binding of M-CSF with the c-fms receptor placed on the surface of presents osteoclast progenitors up-regulates the expression of RANK [11], thus promoting osteoclastogenesis. OPG is the decoy receptor of RANK-L that strongly inhibits osteoclast formation and activity by preventing the interaction with its receptor RANK [12]. The RANK/RANKL/OPG and M-CSF/c-fms systems represent the main mechanisms of coupling between stromal/osteoblastic cells and osteoclast recruitment/activity and play a key role in regulating the balance between bone formation and resorption.

Osteocytes derive from quiescent osteoblasts; they are surrounded by a mineralized matrix and are the terminally differentiated osteolineage cells. Osteocytes, which represent up to 90% of bone cells and have a lifespan of 1 to 50 years [13], respond to mechanical loading and produce various factors involved in the regulation of bone metabolic activities, including RANK-L, Wnt signaling molecules, particularly sclerostin, bone morphogenic proteins (BMPs), nitric oxide (NO) and prostaglandin E2 (PGE2), which modulate osteoclast and osteoblast differentiation and activity. Furthermore, osteocytes modulate phosphate homeostasis through dentin matrix acidic phosphoprotein 1 (DMP1) and fibroblast growth factor 23 (FGF23), and play a key role in bone development and in bone homeostasis, taking an active part in modulating the bone modeling and remodeling processes [1,14].

Bone contains a heterogeneous population of bone marrow cells, including hematopoietic osteoclast precursors and multi-potent BMSCs which can give origin to osteoblasts and to other cell types, such as chondrocytes and adipocytes [15]. BMSCs, that strictly interact with hematopoietic stem cells, have a crucial role in the control and regulation of differentiation, proliferation and metabolism of bone cells, bone remodeling, also influencing osteoblastogenesis, osteoclastogenesis and hematopoiesis [16].

Bone cells respond to mechanical stimulation and to different kinds of local and systemic signals determining the typical bone tissue properties of being plastic and able to adapt to injury, exercise, systemic hormonal and immunological changes, which depend on the intricate relationship among all cellular types present in the bone microenvironment.

## 3. Bone Remodeling and Aging

Bone remodeling is the process that ensures the continuous replacement of damaged and old bone due to the development of load-provoked micro-cracks in bone throughout life and allows the bone to adapt to mechanical changes. Bone remodeling is a result of the close interaction between osteoclasts, that are responsible for the resorption of old or damaged bone and osteoblasts, that are involved in the apposition of new bone tissue. Under normal conditions, during bone remodeling the resorption of old bone is balanced by an equal amount of new bone apposition with a theoretically perfect coupling between the two processes, which ensures the maintenance of stable bone mass. Any pathological or physiological event able to induce an imbalance in bone remodeling phases by increasing bone resorption or by reducing bone formation, or both, can result in bone loss, for which whose main clinical expression is osteoporosis.

Age-dependent changes of bone remodeling have been described. In older individuals there are an increased bone turnover and an imbalance in bone remodeling, mainly due to the reduced osteoblast differentiation and activity. This is usually associated with increased osteoclatogenesis and osteoclast activity, resulting in less new bone formation and increased bone resorption, with consequent bone loss leading to reduced bone mass and increased facture risk [17]. The changes in bone remodeling processes observed with aging can be essentially attributed to an intrinsic alteration of recruitment and activity of bone cells and/or to changes of many different factors that are able to modify bone cells metabolism and/or an altered cellular response to the same factors (Table 1).

## 4. Effect of Aging on Bone Cells

### 4.1. BMSCs

The deterioration of bone strength observed with aging is characterized by the reduction of trabecular and cortical bone density, decreased cortical thickness and increased bone porosity. The main histomorphometric finding of age-related bone loss is the reduction of the mean trabecular thickness, which is a parameter of bone formation and correlates with the decreased osteoblast bone-forming activity [58]. The decline of bone-formation processes depends both on the reduced recruitment of osteoblasts and on the impaired metabolic activity of mature osteoblasts. Several data confirm that aging is characterized by the reduction of bone tissue which is replaced by bone marrow adipose tissue, as a consequence of decreased osteoblastogenesis and a concomitant enhanced adipogenesis from BMSCs [59] (Table 2). It has been shown that the changes in BMSC proliferation and the switch of differentiation toward the adipogenic lineage is one of the main physio-pathological mechanisms of senile osteoporosis [60,61]. BMSCs deriving from elderly subjects present a decline of osteoblastogenesis and a tendency for adipogenic differentiation [62].

The proliferation and differentiation of BMSCs toward osteoblast or adipocyte lineage are regulated by various biological, biochemical and physical factors. Several transcription factors are implicated in the regulation of the differentiation of BMSCs toward the osteogenic or adipogenic lineage, or in determining the intrinsic BMSC senescence. The principal transcription factors that are determinant in osteogenic differentiation are Runx2 and Osterix, while peroxisome proliferator-activated receptor γ (PPARγ) is the master regulator of adipogenesis (Figure 2). The activation of Runx2, also called Core bonding factor-1 (Cbfa1) is among the mail initiation signals for the commitment of BMSCs to osteoblasts as it induces the transcription of different osteogenic genes such as osteocalcin, 1α-collagen, osteopontin, bone sialoprotein [66]. In senescent bone, the expression of Runx2 is markedly reduced, resulting in impaired osteogenesis and reduced bone formation [25]. Osterix acts as an upstream factor of Runx2; in transgenic mice lacking Osterix, osteoblast differentiation is stopped and no bone formation is observed [67]. Another transcription factor playing an important role in the regulation of differentiation processes toward osteoblasts is forkhead transcription factor P (FOXP), whose deficiency in BMSCs is associated with premature bone aging, the reduced number of osteoblasts, increased expression of adipogenic markers and the gradual decrease in bone mineral density (BMD) [26].

The stimulation of BMSCs with sera derived from elderly subjects (used as a bone microenvironment model) reduced the expression of osteoblastic genes Cbfa1/Runx2, alkaline phosphatase (ALP), type I collagen and osteocalcin, resulting in an impaired osteoprogenitor cells recruitment and osteoblast differentiation [68]. BMPs, proteins belonging to the transforming tumor growth factor (TGF)β superfamily, enhance osteogenic differentiation by increasing Runx2 expression, which in turn increases Osterix expression [10]. In a rat animal model, aging is associated with reduced bone forming capacity in response to BMP stimulation [69].

Conversely, the most important transcription factor for the adipogenic differentiation of BMSCs is PPARγ [70]. The up-regulation of PPARγ promotes the differentiation of BMSCs into adipocytes; it has been shown that in older bone the expression of PPARγ is increased [27] leading to enhanced adipogenesis and reduced osteogenesis [25]. Nuclear factor erythroid 2-related factor 2 (NRF2) is another transcription factor whose down-regulation is observed during bone aging and is associated with reduced bone mass and the loss of loading-induced anabolic response [29].

The exact molecular mechanisms underlying the differentiation shift of BMSCs toward the adipogenic lineage to the detriment of osteoblastogenesis are not clearly established [3]. Some studies demonstrated that miRNAs could be involved, as miR188 knockout mice presented less age-related bone loss and increased lower bone marrow fat; conversely, transgenic mice overexpressing miR188 showed accelerated age-related bone loss and the concomitant increase in marrow fat. The bone effects of miR188 are related to histone deacetylase 9 (HDAC9) and the rapamycin-sensitive companion of mammalian target of rapamycin (RICTOR) mRNAs. HDAC9 inhibits adipocytes formation, while favoring osteoblastogenesis through the inactivation of PPARγ—and it is extremely down-regulated during adipogenesis [71]. RICTOR suppresses PPARγ activity and inhibits the adipogenic differentiation of BMSCs [72]. miR-188 post-transcriptionally inhibits HDAC9 and RICTOR expression thus up-regulating PPARγ expression during the age-related adipogenic differentiation of BMSCs [71].

Additionally, Wnt signaling contributes to the osteoblast differentiation of BMSCs. Transgenic Wnt10b-null mice presented an increased trabecular bone mass at 2–4 weeks of age, which was quickly reduced with aging, together with a decreased expression of osteogenic genes in BMSCs; therefore it has been suggested that the increased adipocyte number in aging bone could be related to the reduced activity of Wnt10b [28]. These findings support the hypothesis that the imbalance of transcription factors favoring BMSC differentiation toward the adipogenic lineage, with an associated reduced osteoblast differentiation, plays a crucial role in the modulation of mechanisms underlying the pathogenesis of bone loss related to senescence.

### 4.2. Osteoblasts

Age-related bone changes do not only depend only on the reduced osteoblast differentiation from BMSCs, but they are also associated with the decline of lifespan and the altered metabolic activity of mature osteoblasts [16]. Reduced osteoblast differentiation and the bone-forming capacity of mature osteoblasts have been recognized as the main pathologic mechanisms of low bone mass in elderly subjects and in animal models of aging. Nevertheless, the molecular mechanisms underlying the impaired osteoblast activity are not clearly established. Several in vitro studies revealed that osteoblasts derived from aged donors present an altered phenotype and express lower levels of osteoblastic markers, such as type I collagen and decorin [30,31,32], even though other studies showed conflicting results [73]. Osteoblasts from elderly subjects express higher levels of IL-6, which is a pro-osteoclastogenic interleukin, and lower levels of OPG [64]. Conversely, in human cancellous bone obtained from young individuals, the expression of various osteoblastic bone-forming-related genes, including RUNX-2, Osterix, osteopontin and osteocalcin, as well as the expression of bone resorption markers, such as RANK-L and the matrix metalloproteinase 9 (MMP-9), were significantly higher compared to that of bone obtained from older subjects, indicating a decreased bone remodeling in elderly individuals associated with reduced bone density [74].

The bone-forming ability of osteoblasts also depends on their functional lifespan. At the end of the bone remodeling cycle a great percentage of osteoblasts, estimated to be between 60% and 90%, close their life cycle by apoptosis. The increase in osteoblast apoptosis has been proposed among the mechanisms of the reduced bone-forming capacity of senescent bone [75]. Histomorphometric data suggest that the age-dependent bone loss depends on increased osteoblast and osteocyte apoptosis which can be related to increased oxidative changes [18], however the exact role of osteoblast apoptosis in bone senescence is not clear and available data are discordant [25,76].

With aging, various Wnt proteins and co-receptors are down-regulated, resulting in reduced osteoblast activity and consequent bone loss. Mature osteoblasts differentiated from aged mice show a reduced expression of various Wnt proteins, such as Wnt1, 4, 5a, 5b, 7b, 9b, 10b, and LRP-5, as well as a significantly reduced expression of the Wnt inhibitors Dkk-1 and secreted frizzled-related protein 1 (sFRP1), compared to the osteoblasts derived from younger mice. The reduced bone density in older mice, even with the reduction of both Wnt ligands and Wnt inhibitors, could be explained by an alteration of the ratio of Wnt ligands to Wnt inhibitors characterized by a relative higher expression of Wnt inhibitors leading to reduced osteoblastogenesis and osteoblast activity [28,33].

It has been suggested that senescent osteoblast changes may be related to the intrinsic alterations of the bone microenvironment occurring with aging. Nevertheless, the relationship between the changes found in the bone microenvironment and the age-related bone loss has not been confirmed [77,78].

### 4.3. Osteoclasts

Defective bone formation observed with aging due to the reduced number of osteoblasts is associated with the increased number and activity of osteoclasts thus resulting in negative bone balance [18]. Actually, it has been hypothesized that age could exert opposite effects on osteoclast activity and bone remodeling in cortical and trabecular bone. In an animal model, aging was associated with increased bone porosity due to the enhanced osteoclast activity in cortical bone, whereas the osteoclast number was decreased in trabecular bone, with a concomitant reduction of the bone marrow levels of RANK-L [18,79,80].

In studies performed on animal models, an age-dependent increase in osteoblast-mediated osteoclastogenesis was observed. This phenomenon was associated with the increased expression of RANK-L and M-CSF in stromal/osteoblastic cells in older subjects, while the OPG expression was reduced [19,20]. OPG expression was significantly lower in human BMSCs derived from elderly subjects [21], supporting the hypothesis that aging is associated with a change in the profile of pro-osteoclastogenic factors favoring osteoclast differentiation and activity [63] resulting in increased bone resorption and bone loss. Thus, bone senescence is characterized by a shift in the expression of RANK-L and OPG that induces an increase in osteoclast formation with imbalance between bone resorption and bone formation, consequently leading to accelerated bone loss. In vitro studies performed on human BMSCs demonstrated an age-dependent enhanced secretion of pro-osteoclastogenic cytokines IL-6 and IL-1β [22] and an increased expression of RANK, RANK-L and c-fms [63].

It has been shown that alterations of the bone matrix components occurring with age can be associated with changes in osteoclast differentiation and activity. In the extracellular matrix of old bone, a significant increase in type I collagen β-isomerization of C-telopeptide is observed compared to young bone, associated with an augmented osteoclast number and activity, which was on average 300% higher in aged bone when compared to young bone [23]. These data suggest that osteoclasts preferentially differentiate and increase the resorbing activity in old bone, which is characterized by the molecular changes of matrix components. Nevertheless, other ex vivo studies found opposite results, showing that the bone matrix components of bone specimens obtained from older subjects induced the formation of fewer osteoclasts compared to younger individuals. The different results are probably due to the different experimental conditions [81]; however, taken together these data suggest that aging is associated with the changes of bone matrix components which can contribute to the imbalance of bone remodeling.

### 4.4. Osteocytes

Another typical histologic finding of bone aging is the reduction of the number of osteocytes. Osteocytes are the bone cells responsible for mechano-transduction and the modulation of bone adaptive response to mechanical loading [82]. Moreover, they play a central role in the coordination of mechanical and hormonal factors involved in the regulation of bone mass, through the complex lacuno–canalicular system that permits cell interconnection and communication. The osteocyte lacuno–canalicular system is essential for the flow of canalicular fluid that carries nutrients and signaling factors between osteocytes and from osteocytes to other bone cells, including bone marrow cells. Furthermore, the fluid flowing within the lacuno–canalicular system during mechanical loading represents the central stimulus for osteocytes to mediate mechano-transduction [83]. The reduced physical activity occurring with aging engenders a condition of decreased mechanical loading on bone; as a matter of fact, it has been shown that mechanical unloading in long-term immobilized patients leads to bone loss. The higher sclerostin serum levels found in these patients compared to the controls suggest that sclerostin is involved in disuse osteoporosis in humans, potentially via the Wnt/beta-catenin signaling inhibition [84]. Furthermore, the anabolic response of bone to mechanical loading is progressively impaired with age, participating to the altered bone homeostasis observed with aging. In mice, the mechanical loading threshold for the activation of new bone formation is higher in older animals, suggesting that old bone is less responsive to mechanical stimuli [85].

Several data confirm that osteocytes contribute to age-related bone loss as they are crucial in the maintenance of bone homeostasis through the regulation of metabolism and the activity of osteoclasts and osteoblasts. Osteocytes regulate osteoclast recruitment and activity being the major source of both MCS-F and RANK-L and control osteoblasts activity through the production of Wnt signaling modulators, such as sclerostin [86]. Age-dependent changes in the lacuno–canalicular system underlying the osteocyte network have been described [65]. Histomorphometric studies on human bone showed that lacunar density decreases in a linear manner with age, with a significant reduction ranging from 15 to 30% in aged bone [39,40]. The reduction of osteocyte lacunae has also been confirmed in animal studies [41]. Nevertheless, even if the amount of osteocyte lacunae can reflect osteocyte activity, as the lacunar number probably does not exactly reflect osteocyte changes, as lacunae can be empty, mainly in older bone. A microscopy study performed on iliac crest biopsies from women aged from 20 to 73 years old showed a reduction of osteocyte density other than lacunae density in older subjects [42]; however, the lacunae number was higher compared to the number of osteocytes, confirming that the percentage of empty lacunae increases with aging [41]. These data were also confirmed in animal studies, that showed an increment of empty lacunae in the bone of aged mice by histomorphometric analysis [43]. It is possible to hypothesize that empty lacunae reflect the complete dissolution of osteocyte apoptotic bodies. Morphologic studies in human and mice showed features of osteocyte apoptosis in aged bone, consisting in advanced chromatin condensation, cell shrinkage and fragmented DNA [44]. Connexin 43, a protein involved in mechano-transduction that inhibits osteocyte apoptosis, is reduced with aging [45]. It has been shown that enhanced osteocyte cell death is associated with the increased expression of RANK-L leading to the increased osteoclast recruitment and activity and subsequent activation of bone resorption [87]. Several mechanisms can be involved in the increased osteocyte apoptosis observed with age, including the age dependent increase in reactive oxygen species (ROS) in osteocytes, the increased glucocorticoid circulating levels, and the increased intracellular accumulation of damaged organelles and micro-molecules due to the impaired autophagy processes [40]. These data support the previous observation of reduced osteocyte numbers associated with aging both in human [39] and in mouse models [13]. A relationship between the diminished osteocyte density and viability and the accumulation of bone microdamage has been established, confirming that the impaired response to microdamage in aged bone with the reduction of osteocyte function and/or numbers contributes to the alteration of bone mechanical integrity.

Besides the reduction of osteocyte and lacunae number, a significant age-related reduction of osteocyte dendrites has been described using electron microscopy and histomorphometric analysis, with a linear negative correlation between the dendrite number and age [46]. Conversely, the number of osteocyte dendrites positively correlated with cortical thickness, suggesting that the preservation of the osteocyte network is essential to maintaining bone mass [88]. Since osteocyte dendrites play a key role in mechano-transduction, it has been suggested that the reduction of dendrites may contribute to the attenuated bone anabolic response to mechanical loading observed with aging [47]; the reduced connectivity between osteocytes leads to the impairment of mechano-sensitivity and to altered bone quality.

## 5. Age-Related Changes of Bone Cells to Hormone Response

Bone remodeling is influenced by various humoral factors, which act in an autocrine, paracrine and endocrine manner as a consequence of the close relationship between the different bone cells.

Senescent osteoblasts show an altered response to various hormones and growth factors that are involved in the control of bone formation. Insulin-growth factor-1 (IGF-1) and the growth hormone (GH), whose action is mediated by IGF-1, play a key role in the regulation of the growth, development, metabolic activity and lifespan of several cells and tissues, including bone. IGF-1 is essential in ensuring bone growth during skeletal development, is required to mediate the effect of the parathyroid hormone (PTH) on bone, is involved in BMSC proliferation and differentiation into osteoblasts and also in the regulation of osteoblast metabolic activity. Furthermore, IGF-1 plays a key role in osteoclast differentiation [89]. Aging is characterized by a progressive decline in circulating GH and IGF-1 levels, with a concomitant raise in circulating IGF binding protein levels, which bind to IGF-1 inhibiting its activity; moreover, with aging the cellular response to IGF-1 is reduced. These changes of the GH–IGF axis, along with the reduced osteoblast response to GH and IGF-1, contributes to the age-related bone loss. IGF-1 stimulation of cultured osteoblasts obtained from subjects of different ages enhanced cell proliferation and increased the expression of various extra-cellular matrix proteins such as type I collagen, biglycan, fibronectin and decorin in an age-dependent manner, with osteoblasts derived from younger subjects showing a better response [34,35]. Osteoblastic cells derived from aged human donors exhibit decreased proliferative responses to GH and to the platelet-derived growth factor compared with younger donor cells [36]. Cultured osteoblasts deriving from younger subjects show a greater increase in idroxyproline production in response to estrogen stimulation compared to osteoblasts obtained from older subjects [37]. Many studies proved that 1.25 (OH)Vitamin D_3_ stimulation was less effective in increasing the expression of the osteoblastic markers osteocalcin and ALP in osteoblasts derived from aged subjects [38,73].

Several cytokines are able to modulate osteoblast and osteoclast recruitment and activity both in physiological and pathological conditions. It has been clearly shown that the inflammatory cytokines TNFα, IL-1, IL-6 and IL-17 promote osteoclastogenesis and osteoclast activity and on the other hand they inhibit osteoblast differentiation and function; conversely, interferon (IFN)-β, IFN-γ, IL-4, IL-10, IL-12 inhibit osteoclast function, whereas IL-4 has been reported to stimulate osteoblast proliferation and migration and to inhibit osteoblast differentiation. TGFβ can exert both an inhibitory and a stimulatory effect on osteoclasts and osteoblasts [90]. It has been observed that aging is associated with a two- to four-fold increase in circulating pro-inflammatory cytokines, such as IL-6, TNFα- and ROS, even in the absence of a clinically evident chronic inflammatory disease [24], in response to the antigen exposure throughout life. Cytokines and mediators of this condition of “low-inflammatory” state related to age act by modifying the metabolic activity of bone cells inducing an imbalance in bone remodeling processes favoring bone loss.

## 6. Oxidative Stress and Bone Senescence

The increase in ROS is an important contributing factor in aging related changes in all tissues, including bone. Several clinical and experimental data suggest that the oxidative stress plays a crucial role in determining bone alterations associated with senescence, acting through different mechanisms, including enhanced bone cell apoptosis. In murine models of premature aging and features of oxidative stress, osteoporotic fractures are observed [91]. It has been shown that in estrogen-sufficient female mice the progressive age-dependent bone loss was associated with decreased osteoblast and osteoclast numbers, increased osteoblast and osteocyte apoptosis and a reduced bone formation rate which are related to increased levels of ROS and the enhanced phosphorilation of p53 and p66shc proteins that increase the mitochondrial ROS production and cell apoptosis in mice [18]. The association between the high levels of ROS and decreased BMD, as well as the positive effect of antioxidant on bone resorption, have been confirmed in various human clinical studies [48,49,50,51]. ROS activate the FoxO transcription factors, which in turn activate several genes involved in cell defense mechanisms against oxidative stress. β-catenin, that plays an essential role in Wnt-mediated osteoblast differentiation, is crucial also for the FoxO activation induced by ROS [92]. In a murine model of accelerated senescence, the increase in ROS and the reduced BMD observed with age were associated with an increased expression of FoxO target genes as a defense mechanism and with a concomitant decreased expression of β-catenin/T-cell factor (TCF)-related genes. Increased β-catenin levels antagonize the negative effect of ROS on TCF transcription [52]; indeed, mice overexpressing Wnt10b and LRP5 (β-catenin upstream factors) present increased BMD and no evidence of age-related bone loss [93,94]. Transgenic mice lacking FoxO showed an enhanced osteoblast and osteocyte apoptosis and decreased bone mass related to the increase in ROS, as the rate of apoptosis in ex vivo cultured osteoblasts can be reversed by adding antioxidants. Conversely, transgenic mice overexpressing FoxO showed decreased osteoblast apoptosis, increased osteoblast number and bone formation rate and higher bone mass [13]. The age-related increase in the products of lipid oxidation is associated with the increased expression of PPARγ in mice together with the inhibition of Wnt signaling, leading to increased adipogenesis. Adding oxidized lipids to osteoblast cultures increases oxidative stress, contributing to the switch of β-catenin from T-cell specific transcription factors to FoxO transcription factors, thus reducing the suppressive effect of β-catenin on PPARγ gene expression and enhancing adipogenesis, while attenuating Wnt-mediated osteoblast differentiation and proliferation [18].

## 7. Genetic Damage and Bone Senescence

The causes underlying the age-related dysfunction of bone cells have to date not been identified. Various genetic mechanisms are involved in the intrinsic age-related cellular changes that contribute to determining the senescent alterations which affect bone cells and lead to bone loss. Senescent cells present several genetic alterations, including telomere shortening, the up-regulation of senescence-related genes, impaired DNA repair processes and DNA damage response, and the development of the senescence-associated secretory phenotype (SASP).

Aging is characterized by the accumulation of genetic damage; one of the main mechanisms of cellular deterioration is telomere shortening due to the reduction of telomerase activity. Telomere shortening is involved in the pathogenesis of various age-related diseases and in premature senescence syndromes. In rare genetic diseases such as Werner’s syndrome and Dyskeratosis congenita characterized by clinical features of premature aging, including osteoporotic phenotype with low bone mass, excessive telomere shortening and telomere dysfunction are observed [95,96]. In the normal human population, bone mass correlates with the telomere length of peripheral leukocytes and in older women clinical osteoporosis is associated with shorter telomere length [53]. Conversely, osteoblasts expressing telomerase reverse transcriptase show an increased proliferating capacity, and the transfection of telomerase reverse transcriptase in pre-senescent human osteoblasts prevents bone loss when they are grafted into mice [97].

DNA damage induced by endogenous and exogenous environmental factors represents another crucial mechanism of bone senescence. Human inherited disorders and animal models characterized by altered DNA reparative processes show a clinical phenotype characterized by accelerated senescence and reduced bone mass. Ataxia-telangiectasia-mutated (ATM) gene encodes for a Ser/Thr kinase, which is involved in the reparative processes of DNA double-strand break and when it is inactivated, cells present genomic instability, short telomeres and premature senescence. In a mouse model lacking the ATM gene, decreased bone formation and the defective formation of osteoblasts together with enhanced bone resorption were observed [54]. A mouse model lacking Xpd, a gene crucial for DNA repair, exhibited various features of premature aging, including osteoporosis and kyphosis [55]. These genetic changes were able to induce a DNA damage response, characterized by the enhanced expression of the p53 gene which plays an essential role in regulating cellular DNA repair and is involved in the regulation of the cellular senescence processes, stopping the cellular cycle [56]. A mouse model with the increased activity of p53 shows early the senescence phenotype with reduced bone mass [24].

These genetic alterations are associated with an accumulation of senescent cells related to the creation of an inflammatory microenvironment, that participates in impaired osteoblast function and bone formation [57]. It has been shown that in aged mice several cells of the bone microenvironment, including osteoblast precursors and osteocytes, develop a SASP, that promotes tissue degeneration and is characterized by the enhanced expression of the senescence-associated markers p16Ink4a, p21 and p53. Furthermore, the expression of cytokines that activate and maintain SASP, particularly IL-1α, is increased in the osteocytes of older bone [98].

## 8. Conclusions

Various and complex mechanisms are involved in the pathogenesis of senile osteoporosis which represents a worldwide health concern. Molecular, genetic and intrinsic senescence-related cell alterations contribute to the deterioration of bone mass and quality occurring with age. Even if many data derived from in vitro or animal studies and some reports appear to be conflicting and heterogeneous, probably due to the different experimental approaches, understanding the main physio-pathological processes that underlie bone senescence is essential for the development of new potential therapeutic options for treating age-related bone loss.

## Figures and Tables

**Table 1:** Age-dependent bone loss and its mechanisms.

Age-Related Change		Mechanisms	References
↑ bone resorption	↑ osteoclasts number and activity	↑ expression of RANK-L and M-CSF in OBs	[18,19,20,21,22,23,24]
		↓ expression of OPG in OBs and BMSCs	
		↑ expression of pro-osteoclastogenic cytokines	
		alterations of bone matrix components	
↓ new bone formation	↓ osteogenic differentiation from BMSCs	↓ expression of Runx2	[25,26,27,28]
		↓ expression of FOXP	
		↑ expression of PPARγ	
		↓ activity of Wnt10b	
	↑ osteoblasts apoptosis and ↓ metabolic activity	↓ expression of NRF2	[28,29,30,31,32,33,34,35,36,37,38]
		↓ expression of osteoblastic markers	
		↓ expression of Wnt proteins in OBs	
		↓ levels of IGF-1 and GH	
		↓ OBs response to hormones and growth factors	
↓ bone anabolic response to mechanical loading	↓ osteocytes number and dendrites	↓ expression of Connexin 43	[39,40,41,42,43,44,45,46,47]
	↓ lacunar density	impaired autophagy processes	
↑ cell apoptosis	↑ oxidative stress	↑ levels of ROS	[18,48,49,50,51,52]
		↑ phosphorilation of p53 and p66shc	
		↓ expression of β-catenin/TCF related genes	
accumulation of senescent cells and development of the SASP	Genetic damage	telomere shortening, up-regulation of senescence-related genes, impaired DNA repair processes and DNA damage response	[24,53,54,55,56,57]

RANK-L: receptor activator of NF-κb ligand; M-CSF: macrophage-colony stimulating factor; OBs: osteoblasts; OPG: osteoprotegerin; BMSCs: bone marrow stem cells; Runx2: Runt-related transcription factor 2; FOXP: forkhead transcription factor P; PPARγ: peroxisome proliferator-activated receptor γ; NRF2: nuclear factor erythroid 2-related factor 2; IGF-1: insulin-growth factor-1; GH: growth hormone; ROS: reactive oxygen species; TCF: T-cell factor; SASP: senescence-associated secretory phenotype; ↓: decreased; ↑: increased.

**Table 2:** Effects of aging on bone cells.

Bone Cells	Age-Related Changes	References
BMSCs	↓ osteogenic differentiation	[22,59,62,63]
	↑ adipogenic differentiation	
	↑ secretion of pro-osteoclastogenic cytokines IL-6 and IL-1β	
	↑ expression of RANK, RANK-L	
Osteoblasts	↑ apoptosis	[16,30,31,32,33,35,36,37,47,64]
	↓ metabolic activity	
	↓ bone-forming capacity	
	↓ levels of type I collagen, decorin, OPG	
	↑ levels of IL-6	
	↓ expression of Wnt proteins	
	↓ cellular response to GH, IGF-1, estrogen and 1,25(OH)Vitamin D_3_	
Osteoclasts	↑ number and activity	[18]
Osteocytes	↑ apoptosis	[39,40,41,42,43,65]
	↓ lacunar density	
	↑ empty lacunae	
	↓ osteocyte dendrites	

BMSCs: bone marrow stem cells; RANK: receptor activator of NF-κb; RANK-L: RANK ligand; OPG: osteoprotegerin; GH: growth hormone; IGF-1: insulin-growth factor-1; ↓: decreased; ↑: increased.

## Abbreviations

ALP	alkaline phosphatase
APC	adenomatous polyposis coli
ATM	ataxia-telangiectasia-mutated
BMD	bone mineral density
BMPs	bone morphogenic proteins
BMSCs	bone marrow stem cells
C/EBPα	CCAAT-enhancer binding protein α
Cbfa1	core bonding factor-1
CKI	caseine kinase I
Dkk-1	Dickkopf-1
DMP1	dentin matrix acidicphosphoprotein 1
FGF23	fibroblast growth factor 23
FOXP	forkhead transcription factor P
GH	growth hormone
GSK3β	glycogen synthase kinase-3β
HDAC9	histone deacetylase 9
HSCs	hematopoietic stem cells
IFN	Interferon
IGF-1	insulin-growth factor-1
LRP	low-density lipoprotein receptor related proteins
M-CSF	macrophage-colony stimulating factor
MMP-9	matrix metalloproteinase 9
NO	nitric oxide
NRF2	nuclear factor erythroid 2-related factor 2
OPG	Osteoprotegerin
PGE2	prostaglandin E2
PPARγ	peroxisome proliferator-activated receptor γ
PTH	parathyroid hormone
RANK	receptor activator of NF-κb
RANK-L	RANK ligand
RICTOR	rapamycin-insensitive companion of mammalian target of rapamycin
ROS	reactive oxygen species
Runx2	Runt-related transcription factor 2
SASP	Senescence-associated secretory phenotype
sFRP1	secreted frizzled-related protein 1
TCF	T-cell factor
TGF	tumor growth factor
TNF	tumor necrosis factor
